# Whole Blood Donation Affects the Interpretation of Hemoglobin A_1c_

**DOI:** 10.1371/journal.pone.0170802

**Published:** 2017-01-24

**Authors:** Angelique Dijkstra, Erna Lenters-Westra, Wim de Kort, Arlinke G. Bokhorst, Henk J. G. Bilo, Robbert J. Slingerland, Michel J. Vos

**Affiliations:** 1 Sanquin Blood Bank Division, Zwolle, the Netherlands; 2 Department of Clinical Chemistry, Isala Hospital, Zwolle, the Netherlands; 3 European Reference Laboratory for Glycohemoglobin, Isala Hospital, Zwolle, the Netherlands; 4 Department Donor Studies, Sanquin, Amsterdam, the Netherlands; 5 Academic Medical Center, University of Amsterdam, Amsterdam, the Netherlands; 6 Department Medical Donor Affairs, Sanquin Blood Bank Division, Amsterdam, the Netherlands; 7 Department of Internal Medicine, Isala Hospital, Zwolle, the Netherlands; 8 Department of Internal Medicine, University Medical Center Groningen, Groningen, the Netherlands; Baylor College of Medicine, UNITED STATES

## Abstract

**Introduction:**

Several factors, including changed dynamics of erythrocyte formation and degradation, can influence the degree of hemoglobin A_1c_ (HbA_1c_) formation thereby affecting its use in monitoring diabetes. This study determines the influence of whole blood donation on HbA_1c_ in both non-diabetic blood donors and blood donors with type 2 diabetes.

**Methods:**

In this observational study, 23 non-diabetic blood donors and 21 blood donors with type 2 diabetes donated 475 mL whole blood and were followed prospectively for nine weeks. Each week blood samples were collected and analyzed for changes in HbA_1c_ using three secondary reference measurement procedures.

**Results:**

Twelve non-diabetic blood donors (52.2%) and 10 (58.8%) blood donors with type 2 diabetes had a significant reduction in HbA_1c_ following blood donation (reduction >-4.28%, *P* < 0.05). All non-diabetic blood donors with a normal ferritin concentration predonation had a significant reduction in HbA_1c_. In the non-diabetic group the maximum reduction was -11.9%, in the type 2 diabetes group -12.0%. When eligible to donate again, 52.2% of the non-diabetic blood donors and 41.2% of the blood donors with type 2 diabetes had HbA_1c_ concentrations significantly lower compared to their predonation concentration (reduction >-4.28%, *P* < 0.05).

**Conclusion:**

Patients with type 2 diabetes contributing to whole blood donation programs can be at risk of falsely lowered HbA_1c_. This could lead to a wrong interpretation of their glycemic control by their general practitioner or internist.

## Introduction

Hemoglobin A1c (HbA_1c_) is used worldwide in the diagnosis and monitoring of type 2 diabetes. However, HbA_1c_ can only be interpreted correctly when erythropoiesis and erythrocyte survival are uncompromised. Several conditions such as iron deficiency, (severe) kidney insufficiency and the presence of a hemoglobin variant can lead to an under- or overestimation of HbA_1c_, affecting correct interpretation of glycemic control [1].

When blood donors donate blood they lose approximately one tenth of their blood volume. Consequently they lose one tenth of preformed HbA_1c_. The bone marrow will compensate the blood loss by an increase in erythropoiesis, resulting in an increased flow of newly formed erythrocytes devoid of HbA_1c_. As glycation of hemoglobin is a relatively slow chemical process [2], the increased synthesis of erythrocytes will therefore, theoretically, result in a drop in HbA_1c_ for a number of weeks. Previous research has indeed suggested that blood loss and/or whole blood donation can affect HbA_1c_ formation [3,4]. However, the precise effect of whole blood donation on HbA_1c_ dynamics has not been thoroughly studied and blood donors diagnosed with type 2 diabetes haven’t been included in previous research. In addition, the interplay between iron availability, erythropoiesis and HbA1c following whole blood donation is unknown. Within the Dutch donor population the self-reported incidence of type 2 diabetes is 0.9% [5]. As in most countries volunteers with type 2 diabetes are eligible to donate blood, this could affect medical decision making when diagnosing and/or monitoring blood donors with type 2 diabetes.

## Materials and Methods

### Subjects

Two groups of volunteers at Sanquin, the Dutch national blood service collection center (Zwolle, the Netherlands), were followed prospectively after whole blood donation. For this observational study, one group consisted of 23 non-diabetic blood donating volunteers, the other group consisted of 21 blood donating volunteers with type 2 diabetes. Both groups were matched in age and gender and fulfilled the eligibility criteria for donating whole blood in the Netherlands (Sanquin, Amsterdam, the Netherlands). The volunteers with type 2 diabetes were selected from the Sanquin blood donor database on the basis of their use of diabetes medication. Subsequently, age and gender matched non-diabetic volunteers were selected to reduce bias. The study (NL47160.075.13) was approved by Sanquin’s institutional review board and the Medical Ethics Review Committee (Isala Hospital, Zwolle, the Netherlands). Written informed consent was obtained from all volunteers.

### Procedure

Blood donors were followed prospectively for nine weeks. In the first week they voluntarily donated 475 mL of whole blood and two extra test tubes were collected for determining HbA_1c_ and confounding factors: urea, creatinine, gamma-glutamyl transpeptidase, aspartate transaminase, alanine transaminase, C-reactive protein (CRP) and ferritin. In addition, hematological parameters were determined (hemoglobin, hematocrit, mean corpuscular volume, reticulocytes and reticulocyte hemoglobin content). In the subsequent weeks each blood donor visited the center on the same weekday as the weekday of whole blood donation for collecting two test tubes for determining HbA_1c_, CRP, ferritin and hematological parameters. Vitamin B12 and folic acid were determined predonation and 4 weeks post donation to monitor vitamin status which could affect erythropoiesis. Predonation (baseline) characteristics of HbA_1c_, ferritin, hemoglobin, vitamin B12 and folic acid are listed in Table 1.

**Table 1:** Subject demographics and baseline characteristics.

	Healthy group *mean (sd)*	Diabetes group *mean (sd)*	Reference interval
**Total number of participants**	23	21	
**Age (years)**	60.8 (6.3)	61.7 (6.9)	
**Female (n) / male (n)**	2 / 21	2 / 19	
**BMI (kg/m2)**	27.0 (3.1)	28.6 (3.5)	
**Ethnicity**	100% white	100% white	
**Number of blood donations per donor in 2014**	3.6 (0.79)	2.1 (1.67)	
*Diabetes medication*:			
** metformin (n)**	0	10	
** metformin & sulfonylurea (n)**	0	7	
** metformin & DPP-4 inhibitor (n)**	0	1	
** metformin & DPP-4 inhibitor & sulfonylurea (n)**	0	2	
** metformin & GLP-1 mimetic & sulfonylurea (n)**	0	1	
**Baseline hemoglobin (g/dL) female**	12.9 (0.33)	14.0 (1.37)	12.1–16.1
**Baseline hemoglobin (g/dL) male**	14.5 (0.92)	14.0 (0.80)	13.7–17.7
**Baseline HbA**_**1c**_ **(%)**	5.6 (0.38)	6.8 (0.74)	4.0–6.0
**Baseline HbA**_**1c**_ **(mmol/mol)**	37.9 (4.2)	51.5 (8.0)	20–42
**Baseline ferritin (μg/L) female**	32.5 (16.3)	34 (8.5)	20–150
**Baseline ferritin (μg/L) male**	37.1 (24.3)	58.4 (35.6)	30–350
**Baseline vitamin B12 (pmol/L)**	300 (84.7)	348 (192.7)	150–700
**Baseline folic acid (nmol/L)**	21.5 (7.2)	20.6 (6.4)	5–40

DDP-4: dipeptidyl peptidase-4, GLP-1: glucagon-like peptide-1

Test tubes were labeled anonymously before being sent to the laboratory and were analyzed within three hours at the clinical chemical laboratory of the Isala hospital (Zwolle, the Netherlands). Samples for HbA_1c_ were stored at -80°C and analyzed at the end of the study in one run for each donor to prevent bias. All volunteers had donated previously without any problems. Each week volunteers were asked whether there was any change in diet, medication, health status, infection or other particulars. The study was performed from January until March 2015.

### Laboratory measurements

HbA_1c_ analysis was performed at the European Reference Laboratory for Glycohemoglobin (Zwolle, the Netherlands). HbA_1c_ measurements were performed in duplicate at the end of the study in a single run for each blood donor. HbA_1c_ was measured using 3 different secondary reference measurement procedures (SRMP) certified by the International Federation of Clinical Chemistry and Laboratory Medicine and the National Glycohemoglobin Standardization Program (IFCC and NGSP): Roche Tina-quant HbA_1c_ Gen.2 on Integra 800, immunoassay, IFCC and NGSP certified (Roche Diagnostics, Almere, the Netherlands); Premier Hb9210, affinity chromatography HPLC, IFCC and NGSP certified (Trinity Biotech, Bray, Ireland); Tosoh G8, cation-exchange HPLC, IFCC certified (Tosoh Bioscience, Griesheim, Germany). The SRMP’s have documented good results in the IFCC and NGSP monitoring programs and were calibrated using the IFCC secondary reference material with assigned IFCC and derived NGSP values [6–8].

The analytical coefficients of variation (CV_a_) for the individual analyzers used in this study were determined: Tosoh G8 CV_a_ 0.69% (IFCC) and CV_a_ 0.41% (DCCT); Premier Hb9210 CV_a_ 0.73% (IFCC) and CV_a_ 0.43% (DCCT); Tina-quant CV_a_ 1.91% (IFCC) and CV_a_ 1.11% (DCCT).^9^ Three different SRMP’s were used to show the possible variations between the different analyzers. Hematological parameters were measured using a XN-9000 hematology analyzer (Sysmex, Etten-Leur, the Netherlands). All other parameters were measured using a Cobas 8000 analyzer (Roche Diagnostics, Almere, the Netherlands).

### Data analysis and statistics

Data analysis was performed using Graphpad Prism 6 (GraphPad Software, California, USA). Comparisons between different groups were performed using the two-tailed Mann-Whitney test. *P* values <0.05 were considered significant. Statistical dependence between two variables was assessed using Spearman’s rho. The reference change value ($RCV=2^{1/2}*Z*{\left(CV_{a}^{2}+CV_{w}^{2}\right)}^{1/2}$), used for the assessment of the significance of differences in serial HbA_1c_ results from individual subjects, was calculated using the analytical coefficient of variation and the within-subject biological variation (CV_w_) of each HbA_1c_ assay method mentioned above [9]. The RCV and % HbA_1c_ change or reduction from baseline used throughout the article are based on SI units (mmol/mol) unless stated otherwise. For the non-blood donating group [9] a two-tailed RCV was calculated using a Z value of 1.96 (*P* < 0.05) for interpretation of the change in HbA_1c_. For the non-diabetic group and the group with type 2 diabetes donating whole blood, an one-tailed RCV was calculated using a Z value of 1.65 (*P* < 0.05) for interpretation of the expected reduction in HbA_1c_ [10]. Two volunteers missed one out of 8 follow-up appointments, both appeared on the appointment on the last scheduled visit. Percentage HbA1c decrease was calculated omitting these missing data points, possibly resulting in an underestimation of the maximum HbA1c reduction in two volunteers.

## Results

A total of 44 blood donors (40 male/4 female) participated (Table 1). All blood donors with type 2 diabetes were on metformin medication, 11 blood donors with type 2 diabetes received additional glucose lowering medication (Table 1). Each blood donor with type 2 diabetes reported having a good glycemic control and was not on insulin treatment in accordance with the directive for donating blood in the Netherlands. The presence of comorbidities (e.g. high blood pressure, benign prostatic hypertrophy and others) and additional medicine use (e.g. lisinopril, tamsulosin and others) did meet Sanquin’s eligibility criteria for whole blood donation.

In the course of the study, 16 blood donors reported having symptoms consistent with a common cold, 3 blood donors had symptoms consistent with gastroenteritis and one blood donor suffered from a bronchitis and was the only one receiving antibiotics. Of these in total 20 blood donors, 9 had an elevated CRP (≥5 mg/L). In addition, 2 blood donors had an elevated CRP without medical complaints. During the whole study the maximum CRP found was 35 mg/L. Urea was slightly elevated in 16 blood donors, with a maximum of 10.1 mmol/L (reference range 2.9–7.5 mmol/L). Gamma-glutamyl transpeptidase was elevated in 6 blood donors with a maximum of 97 U/L (reference range <55 U/L). Creatinine, folic acid and aspartate transaminase were normal. One blood donor had an elevated vitamin B12 due to monthly injections (847 pmol/L) and 4 blood donors had a suboptimal vitamin B12 concentration with a minimum of 99 pmol/L (reference range 150–700 pmol/L). Four weeks post donation 3 of these 4 blood donors showed an increase in vitamin B12 concentration within the reference range. Six blood donors had mildly elevated alanine transaminase levels (reference range <45 U/L). Considering the predonation hematological parameters, hemoglobin was slightly below the reference range in 7 blood donors. The lowest hemoglobin measured was 12.3 g/dL in 2 male blood donors (reference range 13.7–17.7 g/dL).

### Effect of whole blood donation on HbA_1c_

**Fig 1:**
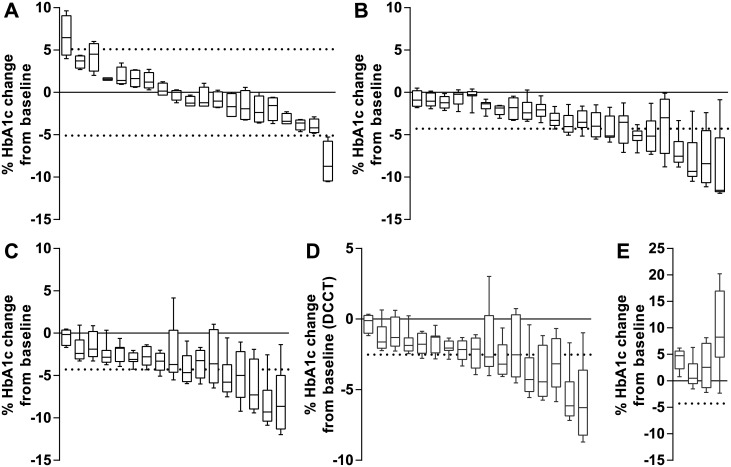
Percentage HbA_1c_ reduction from baseline during 8 weeks post whole blood donation. Data (Tosoh G8) is shown as box plots each representing one individual. Horizontal dotted lines represent the RCV calculated for SI units. Box plots exceeding the RCV represent a significant change or reduction in HbA_1c_ concentration. *A*: Control group of 20 non-diabetic volunteers not donating whole blood.^9^ (Tosoh G8 two-tailed RCV ±5.1%) *B*: 23 non-diabetic blood donors after 475 mL whole blood donation. (Tosoh G8 one-tailed RCV -4.28%) *C*: 17 blood donors with type 2 diabetes after 475 mL whole blood donation. (Tosoh G8 one-tailed RCV -4.28%) *D*: 17 blood donors with type 2 diabetes after 475 mL whole blood donation represented in DCCT units. (Tosoh G8 one-tailed RCV -2.52%) *E*: 4 blood donors with type 2 diabetes with an increase in HbA_1c_ after 475 mL whole blood donation which were excluded from further analysis. (Tosoh G8 one-tailed RCV -4.28%)

Previously, the biological variation of HbA_1c_ in 20 non-blood donating volunteers during a 2 month period was determined [9]. Calculations using data in SI units (mmol/mol) from that study showed that the biological variation in HbA_1c_ varied in both positive and negative direction from baseline with three volunteers exceeding the RCV (Tosoh G8: mean change -0.57% (95% CI -2.71%, 1.57%)) (Fig 1A).

Following whole blood donation, both non-diabetic blood donors (Fig 1B) and blood donors with type 2 diabetes (Fig 1C) showed a reduction in HbA_1c_. Twelve non-diabetic blood donors (52.2%) and 10 blood donors with type 2 diabetes (58.8%) showed a significant reduction exceeding the RCV (Tosoh G8). The other two HbA_1c_ SRMP’s also showed a significant HbA_1c_ reduction in both groups (Premier Hb9210: 13 non-diabetic blood donors (56.5%), 14 blood donors with type 2 diabetes (82.4%); Tina-quant: 8 non-diabetic blood donors (34.8%), 7 blood donors with type 2 diabetes (41,2%) (data not shown)). The mean reduction for the non-diabetic group was -5.45% (95% CI -4.1%, -6.79%) with a maximum of -11.9% for Tosoh G8 (Premier Hb9210: mean reduction -4.73% (95% CI -3.58%, -5.88%), maximum -11.0%; Tina-quant: mean reduction -4.29% (95% CI -2.87%, -5.71%), maximum -9.6%). The mean reduction for the group with type 2 diabetes was -6.0% (95% CI -4.54%, -7.51%) with a maximum of -12.0% for Tosoh G8 (Premier Hb9210: mean reduction -6.23% (95% CI -4.83%, -7.67%), maximum -11.6%; Tina-quant: mean reduction -5.15% (95% CI -3.3%, -6.99%), maximum -16.3%). There was no significant difference in the maximum reduction of HbA_1c_ between non-diabetic blood donors and blood donors with type 2 diabetes. When analyzing the data in DCCT units (%) for blood donors with type 2 diabetes, the reduction in HbA_1c_ was smaller (Tosoh G8: mean reduction -4.08% (95% CI -3.07%, -5.1%), maximum -8.7%) due to the difference between DCCT and IFCC units [11]. More blood donors (13; 76.5%) exceeded the RCV in DCCT units compared to IFCC units (10; 58.8%) (Tosoh G8) (Fig 1D). Four blood donors with type 2 diabetes showed a sharp increase in HbA_1c_ during follow up, probably due to poor glycemic control and where excluded from further analysis (Fig 1E).

### Ferritin status and HbA_1c_

**Fig 2:**
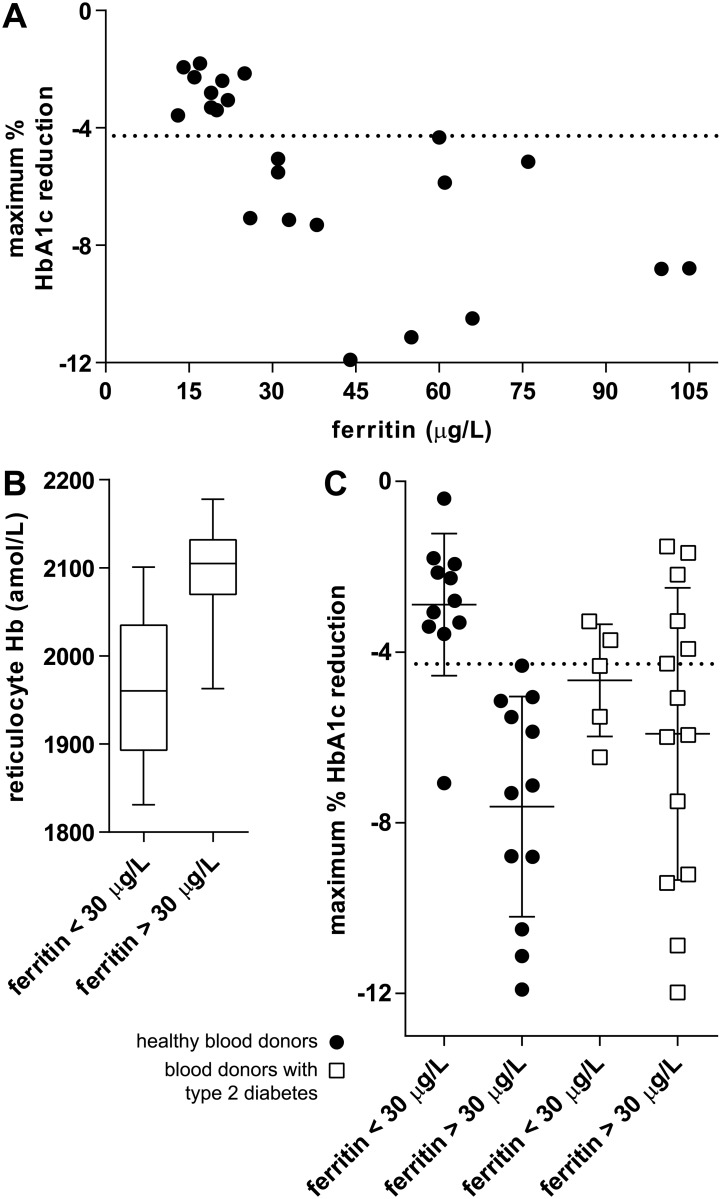
Effect of ferritin concentration on maximum HbA_1c_ reduction (Tosoh G8). Horizontal dotted lines represent the Tosoh G8 one-tailed RCV (-4.28%) calculated for SI units. *A*: 23 non-diabetic blood donors after 475 mL whole blood donation. The predonation ferritin concentration correlated significantly with the maximum % decrease in HbA_1c_ (Pearson’s r = -0.65, *P* = 0.0009). Volunteers without a significant decrease in HbA_1c_ had predonation ferritin concentrations <30 μg/L. *B*: Volunteers with ferritin concentrations <30 μg/L had a significantly lower reticulocyte hemoglobin concentration compared to blood donors with ferritin concentrations >30 μg/L (two-tailed Mann Whitney *P =* 0.0004). Data is presented as box plots. *C*: non-diabetic blood donors and blood donors with type 2 diabetes divided into groups with low and normal predonation ferritin concentration in relation to maximum % decrease HbA_1c_ is plotted. A significant difference in mean maximum % decrease HbA_1c_ between low and normal ferritin concentration was observed for the non-diabetic blood donors (two-tailed Mann Whitney *P* < 0.0001) but not for the blood donors with type 2 diabetes (two-tailed Mann Whitney *P* = 0.69).

A total of 17 blood donors didn’t show a significant reduction in HbA_1c_ (Tosoh G8) after whole blood donation (10 non-diabetic blood donors and 7 blood donors with type 2 diabetes). A possible explanation could be a low ferritin concentration due to the frequency of donation, resulting in a less effective erythropoiesis and as a consequence a reduced effect on HbA_1c_. In the Netherlands, male blood donors are allowed to donate whole blood up to 5 times a year, female blood donors up to 3 times a year. Some of these blood donors develop in time a low ferritin concentration, because of the frequency of donation, and subsequently a low hemoglobin concentration (minimum Hb for whole blood donation: male ≥ 13.5 g/dL, female ≥ 12.6 g/dL) and aren’t allowed to donate blood for at least 3 months. When analyzing ferritin concentrations predonation versus the maximum reduction in HbA_1c_ in non-diabetic blood donors a correlation between both parameters was observed (Pearson’s r = -0.65) (Fig 2A).

Based on this we defined a ferritin concentration <30 μg/L as too low to have a significant lowering effect on HbA_1c_ after whole blood donation. A ferritin concentration <30 μg/L has previously been suggested as a sensitive diagnostic cutoff value for iron deficiency [12]. To further support this cutoff value, the predonation hemoglobin content of reticulocytes (immature erythrocytes) in non-diabetic blood donors was analyzed. Reticulocyte hemoglobin content is a parameter that can be used as an early indicator of functional iron deficiency as it reflects bone marrow iron availability [13]. A significant difference in reticulocyte hemoglobin content was found (*P* = 0.0004) when comparing the low (<30 μg/L) and the normal ferritin (>30 μg/L) group: low ferritin concentration correlated with low reticulocyte hemoglobin content (Fig 2B). The mean reduction in HbA_1c_ of the low and the normal ferritin groups was significantly different for the non-diabetic blood donors (*P* < 0.0001). However, no significant difference in the mean reduction in HbA_1c_ was found between the low and normal ferritin blood donors with type 2 diabetes (*P* = 0.69) (Fig 2C).

### HbA_1c_ dynamics after whole blood donation

**Fig 3:**
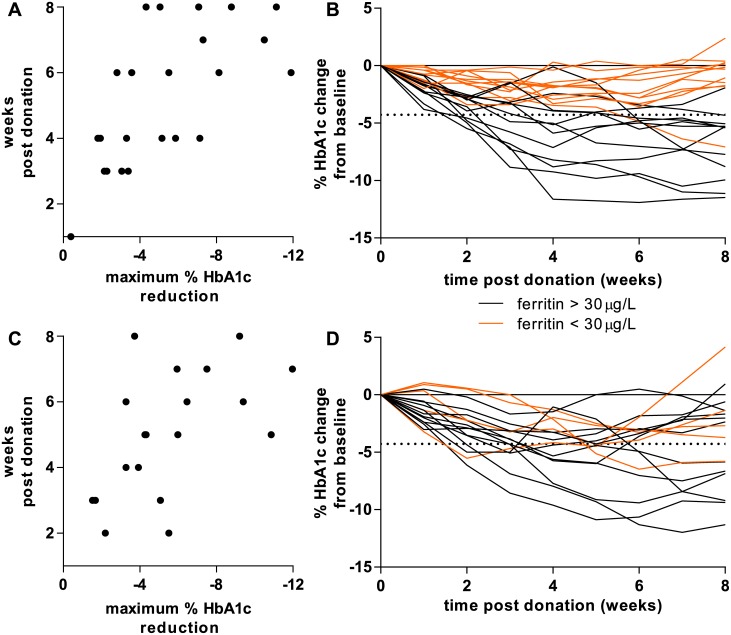
Dynamics of HbA_1c_ during 8 weeks post whole blood donation (Tosoh G8). Horizontal dotted lines represent the Tosoh G8 one-tailed RCV (-4.28%) calculated for SI units. *A*: 23 non-diabetic blood donors after 475 mL whole blood donation. A low maximum % reduction in HbA_1c_ correlated with an early time point in the study, a high maximum % reduction in HbA_1c_ correlated with a late time point (Spearman r = -0.668; *P* = 0.0005). *B*: Individual changes of HbA_1c_ in 23 non-diabetic blood donors. The orange lines represent non-diabetic blood donors with low ferritin (<30 μg/L). *C*: 17 blood donors with type 2 diabetes after 475 mL whole blood donation. A low maximum % reduction in HbA_1c_ correlated with an early time point in the study, a high maximum % reduction in HbA_1c_ correlated with a late time point (Spearman r = -0.547; *P* = 0.015). *D*: Individual changes of HbA_1c_ in 17 blood donors with type 2 diabetes. The orange lines represent blood donors with type 2 diabetes with low ferritin (<30 μg/L).

The above presented data showed that whole blood donation resulted in a significant reduction in HbA_1c_ concentration in more than 50% of the blood donors with a maximum between -9.6% and -16.3% depending on the method used. Knowledge of the time points of maximum HbA_1c_ reduction and normalization are essential for diabetes care. In general, a larger maximum reduction in HbA_1c_ was seen in the last weeks of the study, while a smaller maximum reduction in HbA_1c_ was seen in the first weeks post donation (non-diabetic Spearman’s r = -0.668; type 2 diabetes Spearman’s r = -0.547) (Fig 3A and 3B).

Interestingly, almost none of the blood donors returned to their predonation HbA_1c_ concentration at the end of the study, 8 weeks post donation. Taking the RCV into account, 52.2% of the non-diabetic blood donors and 41.2% of the blood donors with type 2 diabetes didn’t reach their predonation HbA_1c_ concentration (Tosoh G8; Premier Hb9210: non-diabetic 43.5%, type 2 diabetes 52.9%; Tina-quant: non-diabetic 21.7%, type 2 diabetes 11.8%.) (Fig 3C and 3D and data not shown). Almost all blood donors with a low predonation ferritin concentration (Fig 3C and 3D orange lines) did not exceed the HbA_1c_ RCV and as such did not deviate significantly from their predonation HbA_1c_ during the study.

## Discussion

### Statement of principal findings

Patients with type 2 diabetes contributing to whole blood donation programs can be at risk of falsely lowered HbA_1c_ concentrations which could lead to wrong interpretation and therapeutic decision making by their general practitioner or internist. Our study showed that HbA_1c_ dropped significantly after whole blood donation in more than half of the blood donors (Tosoh G8, Premier Hb9210). When correcting for normal ferritin concentrations, all healthy blood donors showed a significant drop in HbA1c. The maximum reductions were dependent on the HbA1c analyzer and assay method used (Tosoh G8 -12%, Premier Hb9210 -11.6%, Tina-quant -16.3%). The differences in observed HbA_1c_ reduction, especially for the Tina-quant, are most likely related to differences in analytical coefficients of variation of the different SRMP’s. There was no difference in relative maximum reduction in HbA_1c_ between non-diabetic blood donors and blood donors with type 2 diabetes. The significant HbA1c reduction observed in our study in all blood donors with a normal ferritin concentration is likely to reflect a general physiological phenomenon and as such would be expected to occur in all whole blood donors.

### Strengths and weaknesses in relation to other studies

Changes in HbA_1c_ after whole blood donation have been studied previously. In 1985 Starkman et al. showed a decrease in HbA_1c_ with a maximal reduction after 4 weeks following blood loss (450 mL) in a small group (n = 12) of non-diabetic volunteers [3]. More recently, a large group (n = 42) of non-diabetic blood donors, who didn’t donate blood for at least 6 months, showed no significant reduction in HbA_1c_ after whole blood donation. However, the time points used for HbA_1c_ measurement were few and mostly short after whole blood donation (500 mL; 24 hours, 1 week, 2 weeks, 3 months) [4]. One study assessed the effect of blood-letting on HbA_1c_ in patients with type 2 diabetes. Blood-letting consisted of three phlebotomies (500 mL each) at a 2-week interval with measurement of HbA_1c_ at 4 and 12 months after the blood-letting sequence. After 4 months a mean decrease of HbA_1c_ of approximately 10% (based on DCCT units) or 15% (based on SI units) was observed [14]. The strength of our study compared to the ones mentioned above is the inclusion of both non-diabetic blood donors and blood donors with type 2 diabetes. In addition we analyzed HbA_1c_ each week for 8 weeks post donation, a time interval after which blood donors are eligible to donate again.

### Unanswered questions and future research

Optimal ferritin status, in combination with vitamin status and kidney function is essential for effective erythropoiesis. It became apparent that non-diabetic blood donors with a normal ferritin concentration predonation had a significantly larger reduction in HbA_1c_ than blood donors with low ferritin concentration. As the effectiveness of erythropoiesis after whole blood donation is dependent on sufficient iron stores, blood donors with normal ferritin concentrations will have a more effective erythropoiesis than blood donors with a low ferritin, therefore leading to a greater reduction in HbA_1c_. As we observed the largest reductions in HbA_1c_ in the second half of this study, the total influx of newly formed erythrocytes seems to be maximal in week 4–8 after whole blood donation. Ferritin concentration is an important predictor of the degree of HbA_1c_ reduction after whole blood donation in non-diabetic blood donors. However, this is not applicable to blood donors with type 2 diabetes. In patients with type 2 diabetes, both newly and previously diagnosed, elevated ferritin concentrations have been reported before [15,16]. Possible explanations for this could be high iron body stores or an acute-phase reaction and therefore may reflect inflammation [15–17]. As there was no significant relation between ferritin concentration and the maximum reduction in HbA_1c_ in blood donors with type 2 diabetes, it is unlikely that elevated iron body stores are the reason for higher ferritin concentrations. This makes it more likely that inflammation is the main cause of higher ferritin concentrations in blood donors with type 2 diabetes. In addition, the number of whole blood donations per year will affect body iron stores. Although non-diabetic blood donors donated significantly more blood one year before the start of this study (Table 1), there was no significant difference between both groups in HbA_1c_ decrease, suggesting that a higher ferritin concentration in blood donors with type 2 diabetes does not reflect the true iron body stores available for erythropoiesis and may even conceal a possible iron deficiency. Taking the other confounding factors into account, these were not associated with changes in HbA_1c_ (data not shown).

Most blood donors didn’t reach their predonation HbA_1c_ concentration at the end of the study. As the study used a follow-up period of 8 weeks, it is not possible to give an advice on when the HbA_1c_ has returned to its predonation concentration and when it can be reliably used by the general practitioner or internist for treatment evaluation or diagnosis of type 2 diabetes. Blood donors are eligible to donate again 8 weeks after the previous donation, with a maximum of 5 times a year for males and 3 times for females. Theoretically this could lead to a sequential reduction of HbA_1c_ resulting in a new steady state which will be lower than the true HbA_1c_ concentration. It should be stressed that reduction of HbA_1c_ due to whole blood donation does not reflect a general decrease in advanced glycation end products and therefore is not associated with a decreased risk of developing complications associated with type 2 diabetes.

### Recommendations

Physicians who are counselling patients with type 2 diabetes who are blood donor or want to become a blood donor, should take into account that blood donation can result in a significant reduction of HbA1c for at least two months after whole blood donation. Therefore we recommend patients with type 2 diabetes who donate blood to have at least a 4 month interval between blood donations. Measurement of HbA1c should preferably be performed at the end of this 4 month interval.

## Supporting Information

S1 Dataset HbA1c blood donation raw data 2017. (XLSX)

## References

[1] Gallagher EJ, Le Roith D, Bloomgarden Z. Review of hemoglobin A(1c) in the management of diabetes. J Diabetes 2009;1:9–17. 10.1111/j.1753-0407.2009.00009.x 20923515

[2] Bunn HF, Haney DN, Kamin S, Gabbay KH, Gallop PM. The biosynthesis of human hemoglobin A1c. Slow glycosylation of hemoglobin in vivo. J Clin Invest. 1976;57:1652–9. 10.1172/JCI108436 932199 PMC436825

[3] Starkman HS, Wacks M, Soeldner JS, Kim A. Effect of acute blood loss on glycosylated hemoglobin determinations in normal subjects. Diabetes Care 1983;6:291–4. 6872811 10.2337/diacare.6.3.291

[4] Borai A, Livingstone C, Farzal A, Baljoon D, Al Sofyani A, Bahijri S, et al Changes in metabolic indices in response to whole blood donation in male subjects with normal glucose tolerance. Clin Biochem. 2016;49:51–6. 10.1016/j.clinbiochem.2015.08.023 26320016

[5] Atsma F, Veldhuizen I, de Vegt F, Doggen C, de Kort W. Cardiovascular and demographic characteristics in whole blood and plasma donors: results from the Donor InSight study. Transfusion 2011;51:412–20. 10.1111/j.1537-2995.2010.02867.x 20804526

[6] IFCC Monitoring Programme [Internet]. IFCC Working Group HbA1c. [cited 2016 Nov 30]. http://www.cuesee.com/Cuesee_frame.asp

[7] Little RR, Rohlfing CL, Wiedmeyer HM, Myers GL, Sacks DB, Goldstein DE. The National Glycohemoglobin Standardization Program (NGSP): a five-year progress report. Clin Chem. 2001;47:1985–92. 11673367

[8] Little RR. Glycated haemoglobin standardization. National Glycohemoglobin Standardization Program (NGSP) perspective. Clin Chem Lab Med. 2003;41:1191–9. 10.1515/CCLM.2003.183 14598869

[9] Lenters-Westra E, Røraas T, Schindhelm RK, Slingerland RJ, Sandberg S. Biological variation of hemoglobin A1c: consequences for diagnosing diabetes mellitus. Clin Chem. 2014;60:1570–2. 10.1373/clinchem.2014.227983 25248570

[10] Fraser CG. Reference change values. Clin Chem Lab Med. 2012;50:807–812. 10.1515/CCLM.2011.733 21958344

[11] Weykamp CW, Mosca A, Gillery P, Panteghini M. The analytical goals for Hemoglobin A1c measurement in IFCC units and National Glycohemoglobin Standardization Program units are different. Clin Chem. 2011;57:1204–06. 10.1373/clinchem.2011.162719 21571810

[12] Muñoz M, Villar I, García-Erce JA. An update on iron physiology. World J Gastroenterol. 2009;15:4617–26. 10.3748/wjg.15.4617 19787824 PMC2754509

[13] Mast AE, Blinder MA, Lu Q, Flax S, Dietzen DJ. Clinical utility of the reticulocyte hemoglobin content in the diagnosis of iron deficiency. Blood 2002;99:1489–91. 11830506 10.1182/blood.v99.4.1489

[14] Fernández-Real JM, Peñarroja G, Castro A, García-Bragado F, Hernández-Aguado I, Ricart W. Blood letting in high-ferritin type 2 diabetes: effects on insulin sensitivity and beta-cell function. Diabetes 2002;51:1000–4. 11916918 10.2337/diabetes.51.4.1000

[15] Ford ES, Cogswell ME. Diabetes and serum ferritin concentration among U.S. adults. Diabetes Care 1999;22:1978–83. 10587829 10.2337/diacare.22.12.1978

[16] Thomas MC, MacIsaac RJ, Tsalamandris C, Jerums G. Elevated iron indices in patients with diabetes. Diabet Med. 2004;21:798–802. 10.1111/j.1464-5491.2004.01196.x 15209778

[17] Hotamisligil GS. Inflammation and metabolic disorders. Nature 2006;444:860–7. 10.1038/nature05485 17167474

